# Risk factor identification and nomogram-based predictive model development for recurrence of pituitary adenomas following transsphenoidal surgery: a dual-center retrospective study

**DOI:** 10.3389/fsurg.2026.1784374

**Published:** 2026-03-27

**Authors:** Xingjian Yang, Wenxian Yang, Pengwei Hou, Yuyang Chen, Jun Li, Jiansheng Zhong, Shousen Wang, Hongjie Chen

**Affiliations:** 1 Department of Neurosurgery, 900th Hospital of PLA Joint Logistic Support Force, Fuzhou, Fujian, China; 2 Department of Neurosurgery, Fujian Provincial Clinical Medical Research Center for Minimally Invasive Diagnosis and Treatment of Neurovascular Diseases, Fuzhou, Fujian, China; 3 Department of Ultrasound, Bengbu Medical University, Bengbu, Anhui, China; 4 Department of Neurosurgery, Jinjiang Municipal Hospital (Shanghai Sixth People’s Hospital Fujian Branch), Jinjiang/Quanzhou, Fujian, China; 5 Department of Neurosurgery, Huashan Hospital Affiliated to Fudan University, Shanghai, China; 6 Department of Neurosurgery, Fujian Medical University, Fuzhou, Fujian, China

**Keywords:** nomograms, pituitary adenomas, prognostic models, transsphenoidal surgery, tumor recurrence

## Abstract

**Objective:**

To identify independent risk factors for pituitary adenoma recurrence after transsphenoidal surgery and construct and validate a nomogram for recurrence risk quantification.

**Methods:**

We retrospectively collected clinical data of 469 patients with pituitary adenomas from Fuzhou 900 Hospital and Huashan Hospital of Fudan University. LASSO regression was applied to screen potential predictive factors, followed by multivariate Logistic regression to identify independent prognostic factors. Nomograms were subsequently constructed to evaluate the model's discriminative ability, calibration, and clinical utility.

**Results:**

Recurrence occurred in 64 cases (13.65%). Multivariate analysis confirmed modified Knosp classification (grades 3B–4), clival invasion, and subtotal resection as independent risk factors (*P* *<* 0.05). The model had AUC 0.905 [95%CI (0.8254, 0.9847)], C-index 0.905, sensitivity 85%, specificity 86.4%, with good calibration and clinical net benefits (DCA: 0.05–0.90 threshold).

**Conclusion:**

The nomogram, which is the first to integrate clival invasion with modified Knosp classification and extent of resection, effectively predicts pituitary adenoma recurrence after transsphenoidal surgery and shows promising clinical utility.

## 1 Introduction

Pituitary adenoma (PA) is one of the common intracranial tumors, accounting for approximately 10%–15% of all intracranial tumors (1, 2). According to the maximum tumor diameter, it can be divided into different types: those with a diameter 1–4 cm are defined as macroadenoma, and those with a diameter >4 cm are defined as giant PA (3, 4). Among them, macroadenomas and giant PAs often grow invasively and are prone to involving surrounding structures of the sellar region, such as the cavernous sinus, sphenoid sinus, clivus, and sellar floor bone (5, 6). The treatment of PA can adopt medication, surgery, and/or radiosurgery, depending on the clinical status and the size of the adenoma at the time of consultation (2). For PA patients with clinical symptoms, surgical resection remains the most commonly used clinical treatment method. However, due to the irregular shape of some adenomas and the involvement of important neurovascular structures, the gross total resection (GTR) rate of PAs is low, and recurrence is common (7). Clinical studies have shown that the postoperative remission rate of pituitary microadenomas (diameter <1 cm) can reach 80%–90%, while that of macroadenomas and giant adenomas is only 40%–70%, and the overall postoperative recurrence risk is as high as 10%–20% (8, 9). Especially when the tumor cannot be completely resected in the first operation due to complex anatomical structures and extensive invasion, the risk of postoperative disease progression or recurrence in patients is significantly increased (10). Therefore, accurate prediction of recurrence risk is crucial for optimizing the diagnosis and treatment path of patients.

In recent years, as an effective tumor prognosis prediction tool, nomogram has played an important role in personalized medicine (11, 12). It can integrate multi-factor information and provide clinicians with an intuitive prognostic evaluation method. Based on this, this study systematically analyze the influencing factors of recurrence of PA patients after transsphenoidal surgery, construct a nomogram prediction model, and comprehensively evaluate its clinical application value in recurrence early warning and postoperative prognosis judgment.

## 2 Materials and methods

### 2.1 General information

This dual-center retrospective analysis included 469 consecutive PA patients who underwent endoscopic transsphenoidal surgery at Fuzhou 900 Hospital and Huashan Hospital of Fudan University between January 2018 and January 2023. All surgeries were performed by senior neurosurgeons with equivalent professional titles at each institution. Inclusion criteria: (1) Age ≥18 years; (2) Complete medical records and imaging data, all of which underwent 3.0 T Magnetic Resonance Imaging (MRI) pituitary thin-slice scanning in our institutions; (3) Postoperatively confirmed as PA by immunohistopathology; (4) Complete clinical and follow-up data. Exclusion criteria: (1) Treated by non-endoscopic transsphenoidal surgery; (2) History of craniocerebral or sellar region surgery; (3) Incomplete postoperative data or loss to follow-up; (4) Preoperative radiotherapy or medication; (5) Complicated with other intracranial tumors or systemic malignant tumors. Patients were reexamined at 3 months, 6 months, and 12 months after surgery, and then annually. The pituitary imaging findings were jointly evaluated by neurosurgeons and neuroradiologists who were blinded to clinical outcomes. Tumor recurrence was defined as: during follow-up, MRI showed a significant increase in residual tumor foci (any diameter >2 mm) or the appearance of new masses (13).

### 2.2 Outcome measures

Follow-up was conducted through telephone, outpatient or inpatient reexamination. According to the recurrence criteria, the study cohort was divided into recurrence group and non-recurrence group. The follow-up cutoff date for all patients was January 1, 2025.

### 2.3 Data collection

① General information: Including age and gender. ② Imaging data: Maximum tumor diameter (MTD): The maximum diameter of the tumor was measured using the Infinitt PACS system. Pituitary adenomas were classified into macroadenomas (1∼4 cm) and giant adenomas (>4 cm) according to diameter (14). Modified Knosp classification: According to the degree of tumor invasion into the cavernous sinus, grades 0–3A were defined as non-invasive, and grades 3B–4 as invasive (15). Hardy–Wilson classification: This classification evaluates the degree of tumor invasion of the diaphragma sellae and suprasellar region (a major type of extrasellar invasion), with grades 1–2 defined as non-invasive (tumor confined to the sella turcica or with mild diaphragma sellae bulging without suprasellar extension) and grades 3–4 as invasive (tumor with obvious suprasellar extension through the diaphragma sellae) (16). Extent of resection: All patients underwent pituitary MRI reexamination 3 to 4 months after surgery, and the residual tumor volume was measured on enhanced images. The extent of resection was divided into GTR (referring to no residual tumor) and subtotal resection (STR, ≥80% of tumor removed) (17). Clinically, tumors with modified Knosp classification grades 3B–4 or clival invasion were more likely to be classified as STR due to anatomical limitations and the risk of damaging adjacent neurovascular structures during surgery. Other characteristics: Record whether the tumor was accompanied by apoplexy or cystic degeneration. ③ Pathological data: Ki-67 index: According to the pathological report, the Ki-67 index was divided into low-level (<3%) group and high-level (≥3%) group (18). Pathological classification: All surgical specimens were examined by the Department of Pathology of our hospital and classified with reference to the classification criteria proposed by the World Health Organization in 2017 and 2022 (2, 19). ④ Biochemical indicators: Pituitary hormone levels of patients before surgery, 1 week after surgery, and 3 months after surgery were collected, including growth hormone (GH), adrenocorticotropic hormone (ACTH), thyroid-stimulating hormone (TSH), prolactin (PRL), follicle-stimulating hormone (FSH), and luteinizing hormone (LH).

### 2.4 Statistical analysis

The data obtained from the research were analyzed using SPSS 27.0 statistical software. Measurement data that conformed to a normal distribution were expressed as mean ± standard deviation. T-tests (for two groups) or analysis of variance (for multiple groups) were used for comparison between groups. Those that did not conform to the normal distribution were expressed as the median [quartile], and comparisons between groups were conducted using the Mann–Whitney U test (two groups) or the Kruskal–Wallis test (multiple groups). Counting data were expressed as the number of cases (percentage), and comparisons between groups were conducted using the *χ*^2^ test or Fisher's exact test. A *P* *<* 0.05 was considered statistically significant.

### 2.5 Model construction and validation

Least Absolute Shrinkage and Selection Operator (LASSO) regression analysis was used to screen the subset of potential predictors of postoperative recurrence of pituitary adenoma. Based on the screening results, multivariate Logistic regression analysis was further used to identify independent predictors of postoperative recurrence. Subsequently, R software (version 4.3.0; R Foundation for Statistical Computing, Vienna, Austria) was used to construct the nomogram prediction model, and Bootstrap method with 1,000 repeated samplings was used for internal validation of the model: The concordance index (C-index) was calculated, and the receiver operating characteristic curve (ROC curve) was drawn and the area under the curve (AUC) was calculated to verify the discrimination of the model; Hosmer–Lemeshow test was used to evaluate the goodness of fit of the model, and the calibration curve was drawn to verify the calibration of the model; in addition, the clinical practical value of the prediction model was comprehensively evaluated by drawing the clinical decision curve (DCA) and Clinical Impact Curve (CIC).

## 3 Results

### 3.1 Comparison of general clinical data

A total of 469 patients were included, including 64 in the recurrence group and 405 in the non-recurrence group. Univariate analysis revealed that there were statistically differences between the two groups in tumor type, modified Knosp classification, clival invasion, sphenoid sinus invasion, extent of resection, preoperative compressive symptoms, and preoperative and postoperative hypopituitarism (*P* *<* 0.05); while there were no significant differences in age, maximum tumor diameter, gender, Hardy classification, cystic degeneration, pituitary apoplexy, tumor immunohistochemical subtype, Ki-67 ≥ 3%, and follow-up time (*P* *>* 0.05, Table 1).

**Table 1:** Comparison of patient characteristic variables among the 2 groups.

Factors	Recurrence (*n* = 64)	Non-recurrence (*n* = 405)	*t/x^2^*	*P*
Age (Y)	49.21 ± 13.20	49.57 ± 13.49	0.058	0.810
MTD (mm)	30.12 ± 8.50	29.28 ± 8.68	0.343	0.559
Gender				
Male	35 (54.69%)	259 (63.95%)	2.027	0.155
Female	29 (45.31%)	146 (36.05%)		
Tumor type				
Macroadenomas	22 (34.38%)	269 (66.42%)	24.099	<0.001
Giant adenomas	42 (65.63%)	136 (33.58%)		
Modified Knosp classification				
0–3A	23 (35.94%)	238 (58.77%)	11.669	<0.001
3B-4	41 (64.06%)	167 (41.23%)		
Hardy–Wilson classification				
1–2	28 (43.75%)	225 (55.56%)	1.027	0.311
3–4	36 (56.25%)	180 (44.44%)		
Clival invasion				
Yes	13 (20.31%)	27 (6.67%)	13.191	<0.001
No	51 (79.69%)	378 (93.33%)		
Sphenoid sinus invasion				
Yes	40 (62.50%)	191 (47.16%)	5.203	0.023
No	24 (37.50%)	214 (52.84%)		
Cystic change				
Yes	30 (46.88%)	180 (44.44%)	0.132	0.716
No	34 (53.12%)	225 (55.56%)		
Pituitary apoplexy				
Yes	24 (37.50%)	155 (38.27%)	0.014	0.906
No	40 (62.50%)	250 (61.73%)		
Resection extent				
GTR	41 (64.06%)	352 (86.91%)	65.957	<0.001
STR	23 (35.94%)	53 (13.09%)		
Pressure symptom				
Yes	36 (56.25%)	170 (41.98%)	4.572	0.033
No	28 (43.75%)	235 (58.02%)		
Pre-op hypopituitarism				
Yes	38 (59.38%)	135 (33.33%)	16.099	<0.001
No	26 (40.62%)	270 (66.67%)		
Post-op hypopituitarism				
Yes	35 (54.69%)	168 (41.48%)	3.926	0.048
No	29 (45.31%)	237 (58.52%)		
Tumor immunohistochemical types				
Gonadotroph type	21 (32.81%)	192 (47.41%)	10.863	0.092
Null cell type	11 (17.19%)	59 (14.57%)		
Prolactin type	18 (28.13%)	99 (24.44%)		
Growth hormone type	9 (14.06%)	38 (9.38%)		
Adrenocorticotrophic hormone type	3 (4.69%)	11 (2.72%)		
Plurihormonal type	2 (3.13%)	5 (1.23%)		
Thyrotroph type	0 (0.0%)	1 (0.25%)		
Ki67 ≥ 3%				
yes	22 (34.38%)	95 (23.46%)	3.519	0.061
no	42 (65.62%)	310 (76.54%)		
Follow-up time (M)	47.85 ± 31.30	47.58 ± 28.83	0.135	0.881

*P* *<* 0.05 statistically significant.

MTD, maximum tumor diameter; GTR, gross total resection; STR, subtotal resection; M, month.

### 3.2 Univariate analysis of pituitary hormone levels

Our study further analyzed the differences in pituitary hormone levels between the recurrence group (*n* = 64) and the non-recurrence group (*n* = 405) on the 7th day before surgery, the 7th day after surgery, and 3 months after surgery, involving 6 types of hormones: GH, ACTH, TSH, PRL, FSH and LH. The results showed that there were no statistically significant differences in pituitary hormone levels between the recurrence group and the non-recurrence group before surgery, in the early postoperative period, and in the mid-postoperative period (all *P* *>* 0.05), suggesting that these hormone levels may not be key factors for predicting postoperative recurrence of pituitary adenoma (Table 2).

**Table 2:** Differences between pre and postoperative pituitary hormone levels in all patients.

Hormone	Time	Recurrence (*n* = 64)	Non-recurrence (*n* = 405)	*t*	*P*
GH, ng/mL	Preoperative Day 7	1.20 ± 5.10	0.72 ± 2.41	0.866	0.387
	Postoperative Day 7	0.68 ± 0.72	0.53 ± 0.67	0.890	0.374
	Postoperative Month 3	0.55 ± 2.20	0.60 ± 2.53	0.134	0.893
ACTH, ng/mL	Preoperative Day 7	27.50 ± 26.80	28.80 ± 27.19	0.238	0.812
	Postoperative Day 7	25.20 ± 45.30	26.88 ± 47.93	0.192	0.848
	Postoperative Month 3	30.80 ± 40.50	31.88 ± 43.10	0.144	0.886
TSH, mIU/L	Preoperative Day 7	2.05 ± 2.95	2.17 ± 3.11	0.250	0.803
	Postoperative Day 7	1.40 ± 2.25	1.45 ± 2.32	0.150	0.881
	Postoperative Month 3	2.06 ± 1.34	2.04 ± 1.33	0.101	0.920
PRL, μg/L	Preoperative Day 7	24.80 ± 60.20	24.56 ± 65.11	0.028	0.978
	Postoperative Day 7	13.80 ± 50.50	13.47 ± 52.00	0.049	0.961
	Postoperative Month 3	14.20 ± 43.80	13.79 ± 42.34	0.068	0.946
FSH, mIU/mL	Preoperative Day 7	10.50 ± 22.30	11.92 ± 24.95	0.405	0.686
	Postoperative Day 7	7.10 ± 12.50	7.32 ± 13.09	0.132	0.895
	Postoperative Month 3	7.08 ± 9.80	7.14 ± 10.49	0.046	0.963
LH, mIU/mL	Preoperative Day 7	2.85 ± 2.20	2.81 ± 2.05	0.146	0.884
	Postoperative Day 7	2.70 ± 2.15	2.68 ± 2.05	0.075	0.940
	Postoperative Month 3	3.10 ± 2.25	3.22 ± 2.30	0.342	0.732

*P* *<* 0.05 statistically significant.

GH, growth hormone; ACTH, adrenocorticotropic hormone; TSH, thyroid-stimulating hormone; PRL, prolactin; FSH, follicle-stimulating hormone; LH, luteinizing hormone. Group comparisons were performed using independent samples t-test.

### 3.3 Selection of risk predictors and multivariate analysis

**Figure 1:**
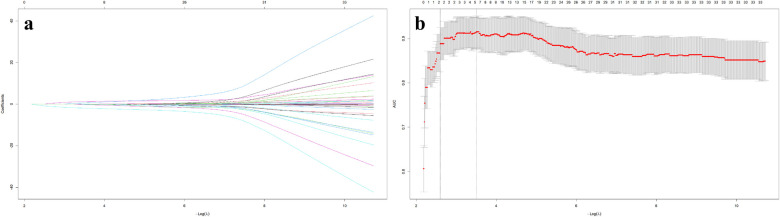
The variables selection using the LASSO logistic regression model. **(a)** Coefficient curves of 34 potential predictive variables. Lasso coefficient profiles of the features. A coefficient profile plot was produced against the log (lambda) sequence. **(b)** A vertical line was drawn at the value selected using tenfold cross-validation, where optimal values by using the minimum criteria and the 1 standard error of the minimum criteria (optimal *λ* = 0.075). The 35 variables included general information (age, gender), imaging data (maximum tumor diameter, tumor type, modified Knosp classification, Hardy–Wilson classification, clival invasion, sphenoid sinus invasion, cystic degeneration, pituitary apoplexy), pathological data (Ki-67 index, tumor immunohistochemical subtype), surgical data (extent of resection), clinical symptoms (preoperative compressive symptoms, preoperative hypopituitarism, postoperative hypopituitarism), and biochemical indicators (growth hormone, adrenocorticotropic hormone, thyroid-stimulating hormone, prolactin, follicle-stimulating hormone, luteinizing hormone at 7 days preoperatively, 7 days postoperatively and 3 months postoperatively). Follow-up time was not included as a potential predictive variable because it represents the duration of outcome observation (for assessing tumor recurrence) rather than a baseline clinical, imaging, pathological, or biochemical characteristic of patients that could predict recurrence risk.

**Figure 2:**
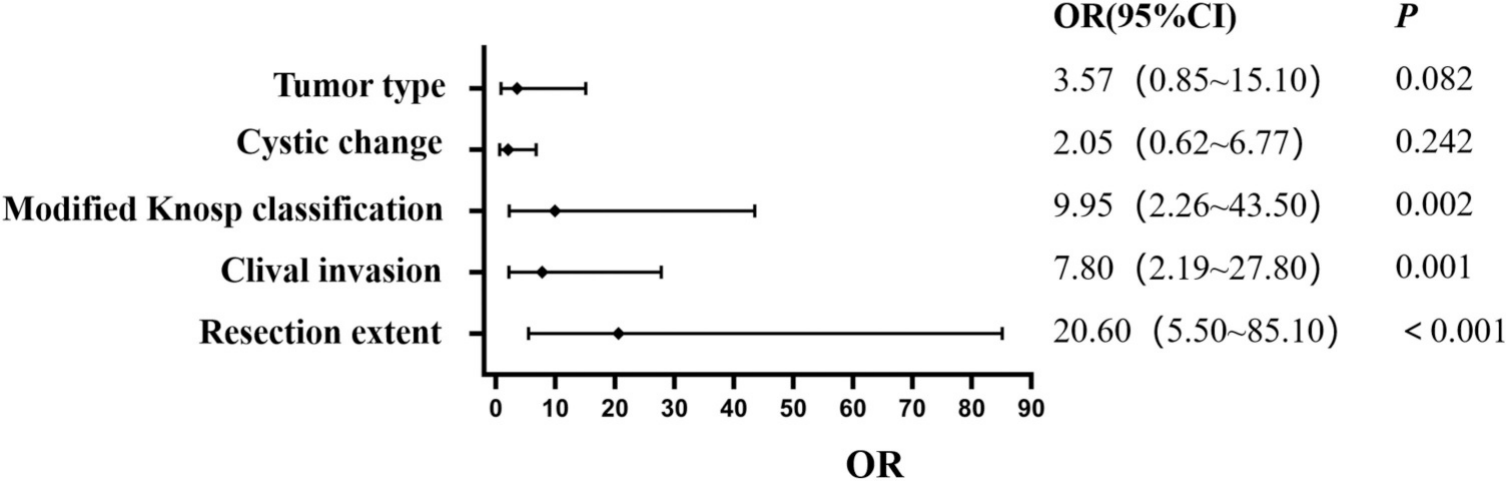
Multifactorial logistic regression scores for predictors of recurrence or progression after transsphenoidal surgery with PA. OR: Odds Ratio is an indicator of the strength of the association between exposure and outcome in observational studies. 95% CI: is the 95% confidence interval for the OR value, indicating that there is a 95% confidence level that the true OR value will fall within this interval in multiple replicate samples.

With postoperative recurrence as the dependent variable, 35 potential predictors were included in the LASSO regression analysis (Figure 1a). In view of the correlation between the respective variables, this study reduced the dimension of the model, and selected the optimal penalty coefficient *λ* (*λ* = 0.075) through 10-fold cross-validation. Finally, five core potential predictors were obtained: tumor type, cystic change, modified Knosp classification, clival invasion and resection extent (Figure 1b). After these five variables were further included in the multivariate logistic regression analysis, the OR of tumor type was 3.57 (95% CI: 0.85∼15.10, *P* *=* 0.082), and the OR of cystic change was 2.05 (95% CI: 0.62∼6.77, *P* *=* 0.242). Both *P* values were > 0.05, which did not reach the statistical significance level, so it was not an independent risk factor for postoperative recurrence. The modified Knosp grade (3B∼4) (OR = 9.95,95% CI: 2.26∼43.50, *P* *=* 0.002), clival invasion (OR = 7.80,95% CI: 2.19∼27.80, *P* *=* 0.001) and STR (OR = 20.60,95% CI: 5.50∼85.10, *P* *<* 0.001) all met the statistical criteria of *P* *<* 0.05, and were independent predictors of recurrence of pituitary adenoma after transsphenoidal surgery (Figure 2). A statistical correlation was observed between these invasive features (modified Knosp 3B-4 and clival invasion) and STR, with 78.0% of tumors with modified Knosp 3B-4 and 84.6% of tumors with clival invasion being classified as STR in our cohort.

### 3.4 Construction of nomogram prediction model

**Figure 3:**
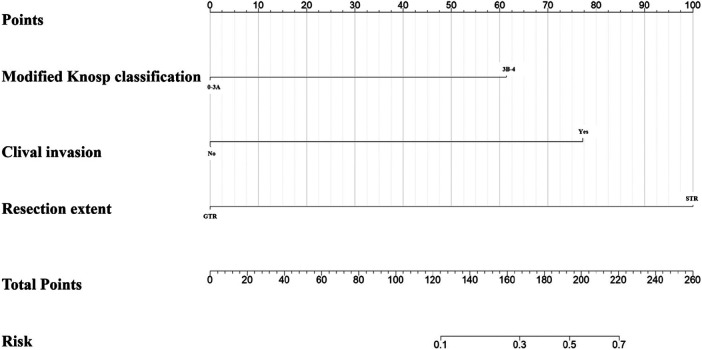
Predicted nomogram of recurrence or progression of PA after transsphenoidal surgery. For each independent predictive factor of the patient, the corresponding individual score can be obtained by projecting the categorical status (0-3A/3B-4 for modified Knosp classification, No/Yes for clival invasion, GTR/STR for resection extent) upward to the uppermost Points scale of the nomogram. The total score of the patient is calculated by summing the individual scores of all three predictors, and then the total score is projected downward to the bottom Risk scale to directly obtain the individualized probability of postoperative recurrence of pituitary adenoma for the patient.

The predictors screened out by the above multivariate Logistic regression analysis were included in the nomogram prediction model. As shown in the figure, the corresponding score can be obtained by projecting the value of each predictor upward to the scale at the top of the nomogram. Then, the total score is obtained by adding the scales of all independent predictors, and the predicted value of postoperative recurrence of pituitary adenoma patients can be obtained (Figure 3).The total score obtained by summing the individual scores of the three predictors corresponds to a specific risk value on the bottom scale of the nomogram, with the risk value ranging from 0.1 to 0.7. This risk value represents the individualized probability of postoperative recurrence of pituitary adenoma, where a value of 0.1 indicates a 10% probability of recurrence, and a value of 0.7 indicates a 70% probability of recurrence; the continuous range of 0.1–0.7 reflects the gradient change of recurrence risk quantified by the model for different patients based on their clinical characteristics.

### 3.5 Efficacy evaluation of the nomogram

**Figure 4:**
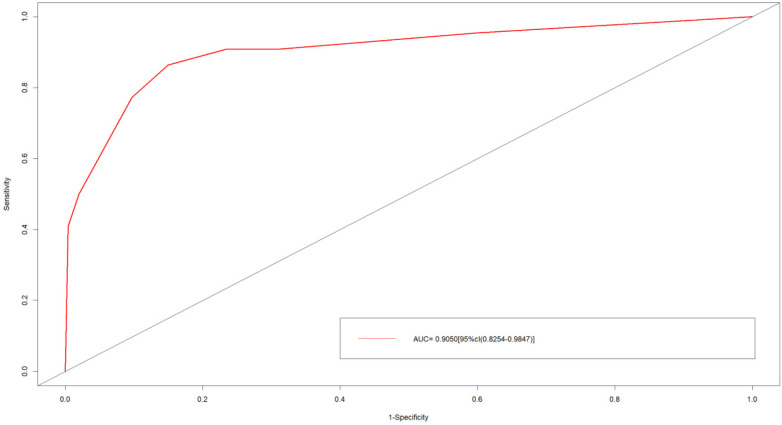
ROC curves of the nomogram prediction model for predicting the recurrence or progression of PA after transsphenoidal surgery. The area under the ROC curve is 0.905.

**Figure 5:**
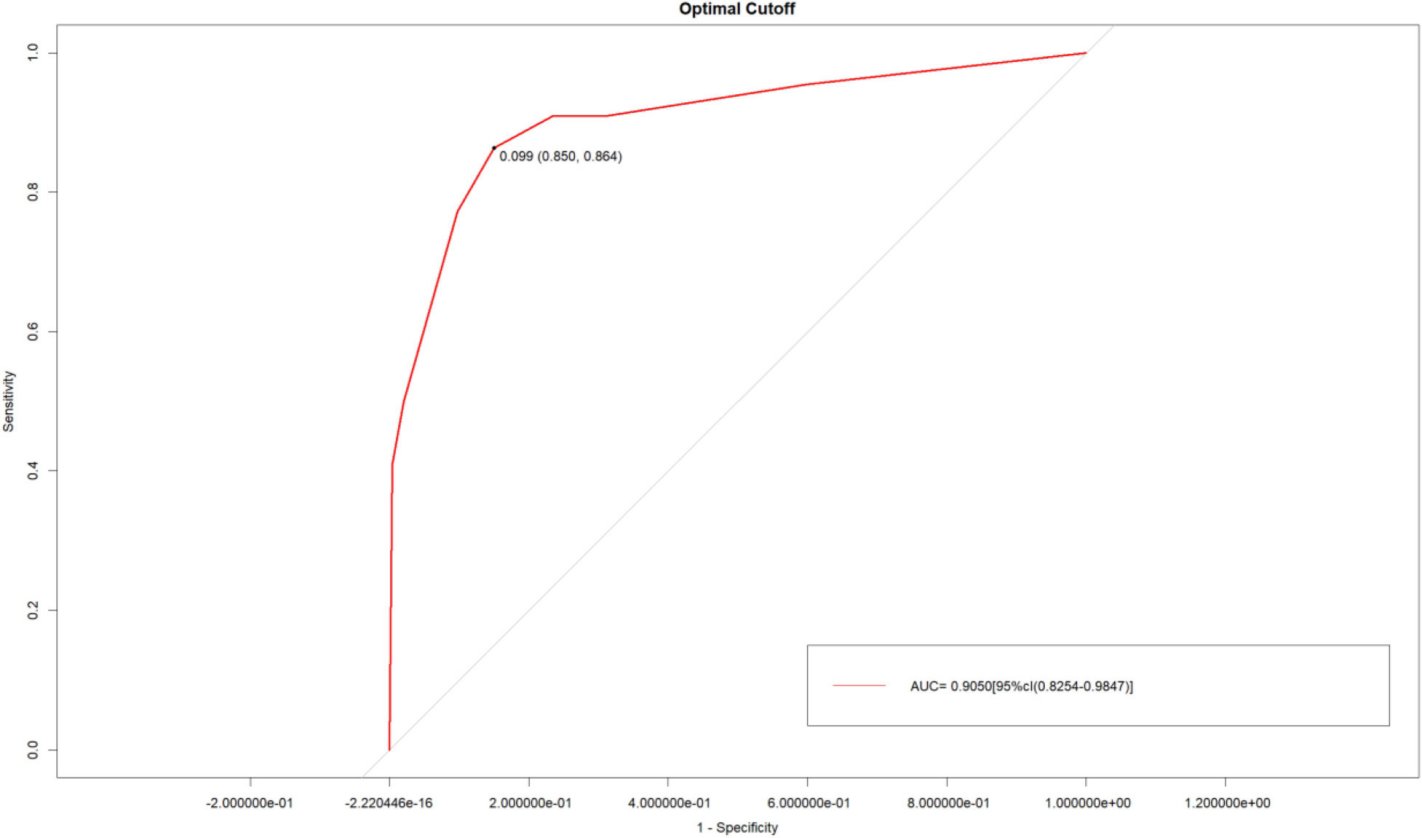
The sensitivity and specificity of the nomogram prediction model for recurrence of pituitary adenoma after transsphenoidal surgery. The sensitivity of this model is 0.850, and the specificity is 0.864.

**Figure 6:**
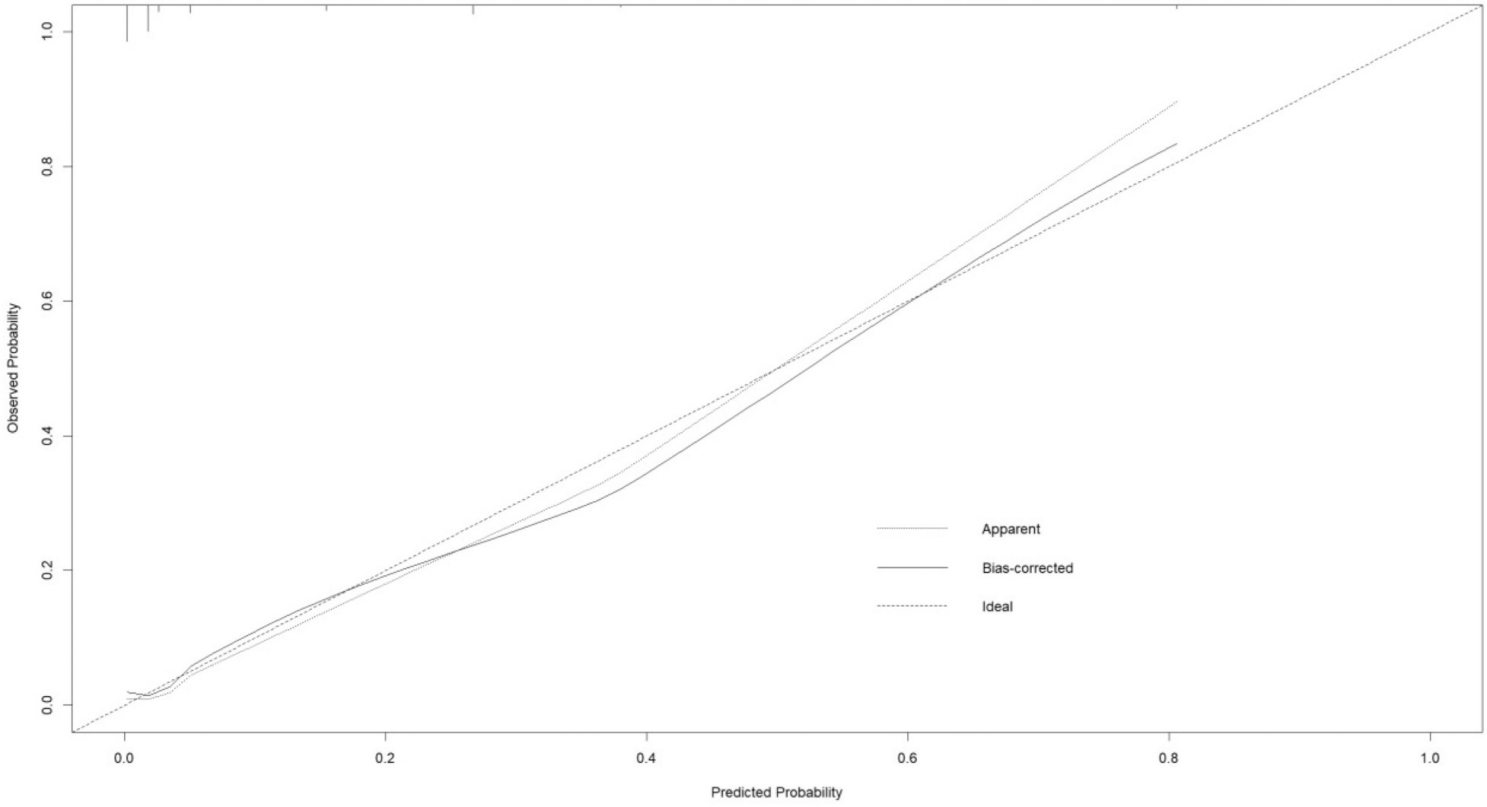
Correction curves for the recurrence or progression of PA after transsphenoidal surgery in the nomogram prediction model. The *x*-axis represents the predicted recurrence risk of PA, and the *y*-axis represents the actual recurrence risk of PA. The diagonal dashed line indicates the perfect prediction of the ideal model. The solid line represents the performance of the nomogram, where closer to the diagonal dashed line indicates a better prediction.

**Figure 7:**
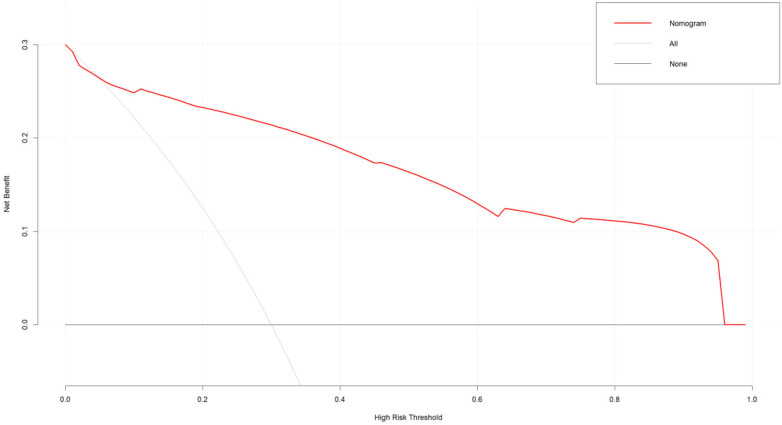
DCA of the nomogram prediction model for predicting the recurrence or progression of PA after transsphenoidal surgery. The *y*-axis represents net benefits. The red line represents the nomogram. The thin solid line indicates the assumption that no patients experience recurrence, and the thick solid line indicates the assumption that all patients experience recurrence.

**Figure 8:**
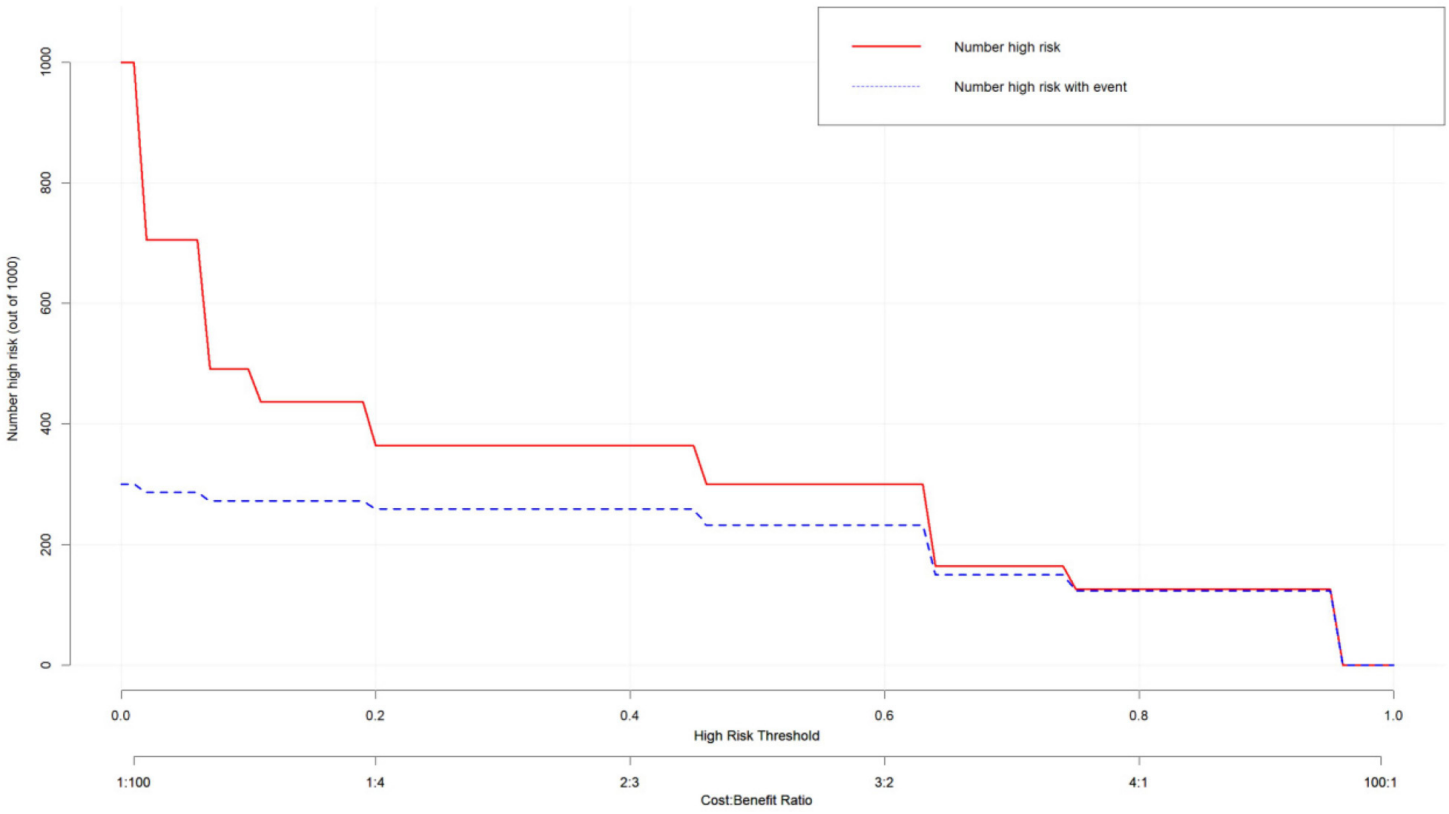
CIC analyses were performed to assess the clinical applicability of the risk prediction nomogram. The *x*-axis is the high risk threshold, the *y*-axis is the number of people at risk in thousands, the red line represents the number of people predicted by the model to experience the ending event, and the blue line represents the actual number of people.

Bootstrap internal validation (1,000 repeated samplings) showed that the C-index of the model was 0.905, the AUC was 0.905 [95% CI: 0.8254–0.9847] (Figure 4), the sensitivity was 85%, and the specificity was 86.4% (Figure 5). The calibration curve showed a high consistency between the predicted risk and the actual risk (Figure 6), and the Hosmer–Lemeshow test indicated a good fit of the model. The DCA curve showed that the model had significant clinical net benefits within the risk threshold range of 0.05–0.90 (Figure 7), and the CIC curve further verified its good identification ability in high-risk populations (Figure 8).

## 4 Discussion

This dual-center retrospective study confirmed that modified Knosp classification (grades 3B–4), clival invasion, and STR are independent risk factors for tumor recurrence. By developing and validating the first nomogram that integrates clival invasion with cavernous sinus invasion (modified Knosp grade) and extent of resection, this study addresses a significant gap in prognostic modeling. This work elevates the level of evidence from single-center, single-factor observations to a validated, multidimensional tool derived from a dual-center cohort. The model demonstrates excellent discrimination, calibration, and clinical utility, providing a practical instrument for individualized risk assessment.

A key contribution of this study is the robust confirmation that clival invasion is an independent and significant prognostic factor. Although previous case reports and imaging series have described clival involvement (often in ectopic adenomas) (20–22), its independent predictive value in a comprehensive multivariate clinical model had not been verified. Our analysis systematically validates this, transforming it from a descriptive or associative imaging finding into a quantifiable and critical prognostic marker. Clival invasion is strongly associated with tumor recurrence due to unique anatomical and surgical challenges. Anatomically, the clivus is a deep midline structure with thin cortical bone and tightly adherent dura mater; the indistinct boundary between the dura and periosteum allows tumors to infiltrate silently along the basilar plexus and petroclival fissure. Surgically, achieving complete tumor resection with clear margins in this region is extremely challenging. The deep, narrow surgical corridor, close proximity to vital neurovascular structures (e.g., basilar artery, brainstem, cranial nerve VI), and the inability to visualize or dissect tumor tissue beyond the invaded bony cortex make GTR risky and often unachievable (23, 24). As a result, residual tumor tissue in the clival bone or dura easily leads to postoperative recurrence.

The prognostic value of cavernous sinus invasion (assessed by modified Knosp classification) is well-established and serves as one of the gold standards for predicting recurrence (10, 25–28). This study not only re-validated it as an important predictor of recurrence, but also highlighted its risk intensity through quantitative OR values: the risk of recurrence in patients with modified Knosp 3B-4 was nearly 10 times higher than that in patients with lower grades. It is worth noting that in the multivariate analysis, the adjusted odds ratio of clival invasion (7.80) was slightly lower than that of modified Knosp 3B-4 (9.95). We speculate that the invasion of the cavernous sinus may more directly reflect the strong invasive biological phenotype of tumor cells breaking through the key neurovascular barrier, and the invasion of the clivus may more directly reflect the incurable surgery caused by the deep anatomical limitations of the skull base. The statistically strong independent correlation between the two shows that they are complementary rather than mutually exclusive risk dimensions. Therefore, incorporating both into the prediction model is essential for a comprehensive assessment of tumor invasion behavior and recurrence risk.

As a key surgical predictor, STR is closely associated with tumor invasive features (modified Knosp 3B-4 and clival invasion) and remains a cornerstone among established risk factors for tumor recurrence (29, 30), a principle robustly reaffirmed by the findings of this study. Notably, within our predictive model, STR emerged not merely as a risk factor but as the most potent independent predictor, exhibiting the highest odds ratio among all variables. This underscores that while tumor biology (reflected by invasion grades) sets the stage for potential recurrence, the extent of surgical resection is the decisive moderating variable that most directly translates biological aggression into clinical outcome. Consequently, the pursuit of maximal safe resection continues to be the paramount objective in surgical strategy (31, 32). This endeavor is particularly critical in cases already burdened by invasive features, where STR would substantially compound the inherent risk. In such scenarios, the nomogram provides a quantitative estimate of this compounded risk, potentially supporting earlier consideration of adjuvant radiotherapy or more vigilant follow-up protocols to mitigate the consequences of residual disease.

It is worth noting that some variables that were significant in the univariate analysis, such as pre-op/post-op hypopituitarism, pressure symptom, and sphenoid sinus invasion, were not screened as the final predictors by the LASSO regression model. This phenomenon may be due to the combined effect of statistical methodology and clinical pathophysiological background. LASSO regression deals with multicollinearity through penalty coefficients while selecting variables. In this cohort, these clinical variables are likely to overlap with the final retained, more powerful anatomical and surgical predictors (i.e., modified Knosp classification, clival invasion, and extent of resection). For example, sphenoid sinus invasion often occurs simultaneously with extensive skull base invasion (including clival invasion) (33), so the prognostic information it provides may not exceed the latter. Similarly, hypopituitarism and pressure symptoms are often the clinical manifestations of large invasive tumors, and their predictive signals may have been covered by more direct indicators of tumor invasiveness and resectability (34). In addition, although giant adenomas are often considered as risk factors for recurrence due to their volume and invasiveness, they are not independent predictors in our multivariate model. Similarly, the Hardy–Wilson classification (suprasellar invasion) was not associated with recurrence, as suprasellar extension is often manageable with surgical resection and does not lead to residual tumor as easily as cavernous sinus or clival invasion. This may be because tumor size, especially the classification of giant adenomas, is often an alternative indicator of invasive behavior and surgical complexity. When more specific invasive direct indicators (such as cavernous sinus and clival invasion) and surgical results (extent of resection) were included in the model, they captured core risk information more accurately. The impact of giant adenomas is largely mediated by these pathways: the larger the tumor, the more likely it is to invade key structures and the more difficult it is to be completely removed. Therefore, when these direct mediating factors enter the model, the independent contribution of tumor size itself is no longer significant. This emphasizes that in the risk stratification of recurrence, the use of accurate anatomical invasion criteria is more advantageous than relying solely on tumor size classification. The LASSO algorithm finally selects the most concise and non-redundant set of predictors, which highlights the characteristics of the model's pursuit of simplicity and prediction performance balance.

A primary objective of this study was to translate statistical associations into a practical clinical tool. The developed nomogram achieves this by providing a visual, quantitative tool suitable for bedside or outpatient use. By summing the points corresponding to a patient's modified Knosp grade, clival invasion status, and extent of resection, clinicians can quickly estimate an individualized recurrence probability. This transforms complex analysis into actionable clinical insights, directly guiding decisions regarding postoperative follow-up intensity and consideration of adjuvant therapy. The model's strong discriminatory performance (AUC 0.905) and its demonstrated net clinical benefit across a wide range of decision thresholds jointly affirm its potential utility in real-world management. The dual-center design and cohort size enhance the methodological rigor and generalizability of the findings, help mitigate single-institution bias, and make the model more robust than those derived from single-center studies.

This study has several limitations. First, although the model was developed using a dual-center cohort, external validation in broader, multi-ethnic populations is warranted. Second, the median follow-up time, though substantial, may not capture very late recurrences. Finally, the model does not yet incorporate molecular biomarkers, which may offer additional prognostic precision.

## 5 Conclusion

In conclusion, this study 1) confirms the independent prognostic value of clival invasion, elevating it from a descriptive finding to a validated risk factor; 2) constructs and validates a novel nomogram that uniquely integrates bidirectional tumor invasiveness (cavernous sinus and clival) with surgical resectability into a streamlined predictive system; 3) translates this integrated model into a clinically practical tool for individualized risk quantification; and 4) provides a higher level of evidence through development and internal validation using a dual-center cohort. This tool facilitates personalized postoperative management strategies, and its prospective validation is recommended for future clinical implementation.

## Data Availability

The raw data supporting the conclusions of this article will be made available by the authors, without undue reservation.
